# The Royal British College of Nursing Born

**Published:** 1917-01-27

**Authors:** 


### THE ROYAL BRITISH NURSES' ASSOCIATION.

### THE ROYAL BRITISH COLLEGE OF NURSING BORN.

A special general meeting of the Royal British Nurses' Association was held in the lecture hall of the Royal Society of Medicine, 1 Wimpole Street, W., on Thursday, January 18, to consider the proposed amalgamation of the College of Nursing, Limited, with the Association.

Amongst those present were Dr. Bezly Thorne, M.D., M.R.C.P. (in the chair), Sir James Crichton-Browne, M.D., F.R.S. Lond. and Edin., Hon. Arthur Stanley, C.B., M.P., Mr. Comyns Berkeley, M.C., M.D., Professor Glaister, M.D., Mr. Herbert Paterson, M.C., M.A., F.R.C.S., Mrs. Latter (late Matron Chelsea Infirmary); Dr. Stark Currie, M.D.; Dr. Walter Griffith, M.D., F.R.C.P.; Dr. Percival White; Mr. T. Mark Hovell, F.R.C.S.; Dr. Howard Barrett; Mrs. Dacre Craven; Miss Sidney Browne, R.R.C. (Matron-in-Chief T.F.N.S.); Miss Melrose, R.R.C., Matron Glasgow Royal Infirmary; Miss Graham, Superintendent Scottish Association of Trained Nurses; Miss E. Montgomery Wilson, R.R.C., Matron King Edward VII. Infirmary, Cardiff; Miss Bickerton, Matron Prince of Wales' General Hospital, Tottenham; Miss Richardson, Matron London Temperance Hospital; Mrs. Bedford Fenwick; Miss Beatty; Miss Cutler, assistant matron, and Miss Marcon, superintendent of private staff, St. Bartholomew's Hospital; Miss Leigh, Matron Endsleigh Palace Hospital; Miss Stewart, Matron City of London Infirmary; and Miss Scott, Matron Royal Sussex County Hospital.

### H.R.H. the President's Letter.

During the meeting Dr. Bezly Thorne, the Chairman, read the following letter:—

"It is a matter of infinite regret to me that circumstances beyond my control prevent my being among you to-day and attending this most important meeting of our Corporation, on the issue of which its future welfare so largely depends. I need not tell you that the question of the amalgamation of the College of Nursing with our Association under the powers conferred on us by Royal Charter has had my most earnest and anxious consideration ever since the project came into existence. Having now looked at it from every point of view and weighed its advantages and disadvantages, I have come to the definite conclusion that the fusion of the two bodies can and will be of great advantage to both the nursing profession at large and the public generally. (Hear, hear.) As regards ourselves, it will bring us an accession of strength and widen our sphere of influence. I would like to call your special attention to the fact that as soon as the united body has passed through the necessary transition stage, its constitution will invest the nurses themselves with power to elect their governing body, and so place in their own hands their own destiny as a profession. (Hear, hear.) You have, ever since the formation of our Association, shown me, and supported me with, the most unshakable loyalty and confidence, and, I may add, affection. For this I thank you from my heart, for I both value and appreciate them warmly. It is with the knowledge of this trust you repose in me that I now ask you to join with me in approving a step which, for the reasons I have stated, will enhance our dignity and importance, and through us confer a boon on the public. (Signed) Helena, President of the Royal British Nurses' Association."

Dr. Bezly Thorne, in opening the proceedings, said that the resolution before the meeting that afternoon embodied the most important question submitted since the foundation of the Association thirty years ago. The fusion of the two bodies would be for the benefit of both, and would have the effect of enhancing the dignity and increasing the efficiency of the nursing profession, while it would be to the great advantage of the public at large.

### A Clear Statement by Mr. Paterson.

Mr. Herbert Paterson, F.R.C.S. (the Hon. Medical Secretary), in proposing the resolution that "The agreement dated December 21, 1916, for the amalgamation of the College of Nursing, Limited, with this Association be confirmed, and that, subject to new by-laws being approved by the Privy Council, the by-laws of June 10, 1898, be annulled," said that at first the scheme of amalgamation was regarded with some suspicion by the Association's Council. Since then the scheme had been remodelled, the orignal germ had been watered, garnished, and pruned, and had blossomed into a ripe and luscious fruit of a somewhat different species from the seed from which it had grown. If the members plucked the fruit presented for their acceptance that afternoon the result should be to found a powerful body which would make its influence felt for the good of the nursing profession. The objection raised in some quarters as to lay influence no longer held good, as out of thirty-nine members of the proposed Council only two were laymen, and in a Council such as this the presence of a few laymen was a decided acquisition. The profession owed a debt of real gratitude to the Hon. Arthur Stanley for taking up this matter, and for the skill and diplomacy with which he had carried it through in spite of opposition and the pressure of other occupations.

The Council of the Royal British College of Nursing would be a democratic one in every sense. If not, the fault would be that of the members, for every member of the Corporation would have a vote. Further, in connection with the power to remove a nurse from the Register the scheme now provided that before this could be done the nurse had the right to appear before the Council, either in person or by agent, and a vote of a majority of at least two-thirds of the members of the Council was required to remove her name.

The only point to consider was, Is the amalgamation for the ultimate good of the nursing profession? The objects of the two bodies were the same—*i.e.*, the establishment of a uniform curriculum; a one-portal examination; State Registration. The amalgamation would materially assist in attaining these objects, and would secure to British nurses a real College of Nursing with the power of self-defence, statutory recognition, State Registration, and, he hoped, a registered uniform or distinguishing badge for fully trained nurses. State Registration was a reform to which the nursing profession was entitled as an act of elementary justice, which Parliament must be induced to grant without delay. Their motto in regard to this should be not "Wait and See" but "Demand and Get." The advisability of this amalgamation had been under consideration for many months, and with the approval of H.R.H. the President, who had consented to be the first President of the new College, the Council and Executive Committee of the R.B.N.A. had unanimously voted in favour of the amalgamation. The Association had got all they asked for. In respect to representation on the new Council they had thirteen members, including Dr. Bezly Thorne (who would be the Vice-President), and Mr. Comyns Berkeley, Hon. Treasurer. Mr. Pitt, their solicitor, would continue as the solicitor to the new Corporation, and Miss Macdonald, their Secretary, would be the Registrar of the Royal British College of Nursing.

Mr. Paterson concluded by saying that from a purely personal point of view the passing of the Association was to him a matter of sincere regret, in that he would no longer be their honorary medical secretary. He hoped as a member of the Council of the new body it might be possible for him to do more in the future than he had always been able to do in the past. He was proud of his association with the R.B.N.A., and it would in the

### THE ROYAL BRITISH NURSES' ASSOCIATION—(*continued*).

future be his earnest endeavour to do all in his power to improve the status, promote the welfare, and further the interests not only of the members of the Royal British College of Nursing but of the whole noble army of trained British nurses.

MRS. LATTER'S CONGRATULATIONS.

Mrs. LATTER, in seconding the resolution, said in her view the Council were to be congratulated on the union. In all Associations there came a period when they had either to go forward or fall back. That period had been reached in the affairs of the R.B.N.A. With the increased powers conferred by the Supplemental Charter the objects for which the Association was founded would be extended and the consolidation of the profession secured. She would like to say one word of comfort and assurance to those of the members who were inclined to view with regret what they seemed to regard as the passing of the R.B.N.A. The only passing would be from a lesser to a greater degree of activity because of the enlargement of their lines and the extension of their powers. They brought to the partnership a fair name and an honourable record, coupled with a determination to remain steadfast in the principles which they had advocated for over a quarter of a century, and true to the best interests of the nursing profession.

THE MAGNA CHARTA OF THE NURSING PROFESSION.

Sir JAMES CRICHTON-BROWNE urged the members to take a broad, comprehensive, and patriotic view of the question submitted to them. The questions to ask themselves were, Is this amalgamation for the benefit of the public? Is it likely to conduce to the welfare, the healing, the relief, of the sick and suffering, to whom it was the nurse's proud privilege to minister? He thought it was. It could scarcely be doubted that one strong, central, well-organised authority representative of the nursing profession in all its branches would secure a uniformity and a degree of excellence that could never be attained when there were a number of independent and rival institutions regulating the profession, and perhaps lowering its standards. The nursing profession was now ripe for a similar change to that which occurred seventy years ago in the medical profession. The beneficent effects of such a change would be felt in many a sick-room and many a hospital and infirmary ward. Enormous strides had been made since the first seeds of modern nursing were sown in the Crimean days; but there was still much to be done, and he believed the finishing touch would be given by the amalgamation they were there to sanction that day. Let them amalgamate and they would get State Registration. Reject amalgamation, and he predicted they would not get State Registration for a very long time to come. State Registration was peculiarly necessary now to distinguish the genuine from the spurious article. Most of the previous Registration Bills had failed because they had not behind them unity and authority. By the fusion of the two bodies referred to in the resolution that weight and impetus would be given. The amalgamation would further secure the building of a College in London as the headquarters of the nursing profession. He looked forward to a time when a stately edifice would proclaim the solidarity of the profession. Let them present a united front, and endowments would flow in to enable the system of education to be perfected and carried out, and many projects for the benefit of nurses to be evolved. In his view there were no nobler or finer specimens of womanhood than true nurses, and he would earnestly exhort the members present to pass the resolution and convert the Charter of the R.B.N.A. into the Magna Charta of the nursing profession in this country.

PROFESSOR GLAISTER AT THE WEDDING.

Professor GLAISTER, who remarked he had come from the North to assist at the wedding ceremony, said that having been working, along with others, in connection with the movement towards the State Registration of Nurses, he saw in this movement of to-day an opportunity of achieving that end which had not hitherto been in sight. During the last twenty years keen, incisive efforts had been made towards State Registration, but, unfortunately, up till now these efforts had not proved successful. To-day they were met together to see the union of two large bodies which could promise to achieve in the near future State recognition and State Registration.

He had been a humble lieutenant of Mr. Stanley in this movement, and a co-worker with others also. He felt the honour of having been associated with both, and thought if all were sincere in the object of securing the State recognition and Registration of nurses they would all go forward unanimously, knowing that where there are many men there are many minds, but that if they had that point in view surely they could let details go, and press forward towards the mark—the mark of Registration and recognition which was desirable at the present moment for the welfare of nursing in this country.

THE MEMBER FROM LAND'S END.

Mrs. MYATT said on hearing of this meeting to-day she felt that neither time nor money must stand in the way of her attending, because, as a member of the Royal British Nurses' Association, she felt her responsibility towards other nurses. Mr. Paterson had told them that they had not very much business capability, but if they could not think for themselves they could think for each other, and so she came from a place a few miles from Land's End to support the meeting. She wished to thank very much the Hon. Arthur Stanley, on behalf of herself and the nurses of her private staff, for the wonderful amount of labour he had put, and was still putting, into this movement.

MISS BEATTY'S REAPPEARANCE.

Miss BEATTY said she wanted to say a few words from rather a different point of view as an old member of the Royal British Nurses' Association. They were practically told this afternoon that they were a failure, and that, therefore, they had to amalgamate with another body to give them strength. What had been the cause of the failure? (Interruption.) She had had experience of the Royal British Nurses' Association from the first beginning, and had stood by it. The cause of their failure had been that the nurses had not the power to study their own interest in this Association. They were formed to work out the interest of the nursing profession. They had had a specimen of it this afternoon. Only one nurse, besides the Secretary, had been allowed to speak, and that was one chosen by the Council. They had no nurses on the platform; they had no voice, and were not allowed to write to their own Journal, which was called the *Royal British Nurses' Journal.* (Interruption and hisses.) She was going to give her opinion of this Royal British Nurses' Association which they were told this afternoon was deceased practically. She was heartily sorry that an Association which was, when she first joined it, in as flourishing a condition as any Association of women could possibly be should come to an end. They were the only body of working women who had secured a Royal Charter. And by whose aid? By the aid of Mrs. Bedford Fenwick and Dr. Bedford Fenwick. It was as flourishing an Association as could possibly have been found in the whole of England, by women, for women, for their interests; and it was only as she told Dr. Bedford Fenwick at the first meeting at Chandos Street—(interruption)—"If you once admit the medical profession into the nursing profession we are going to be swamped." And they had been, because no nurse was allowed to raise her voice in this Association except nurses who were working with certain of the doctors. (Cries of "Sit down!") It was no good telling her to sit down.

A MEMBER said they were quite free to give their opinion.

Miss BEATTY: They might have their own opinion, but she had the floor, and she was entitled to speak. (Cries of

"Sit down!") She had just come from the office of the Royal British Nurses' Association, where, at one time, they were able to pay £700 a year for clerks, office rent, and so on; now she found one servant there who grumbled because she had to give information as to where the meeting was to be held. (Laughter.) Now, as had been rightly stated in some of the journals, they were going to ask lay people on the Council to help to manage it. She happened to hold a very important position just now, where she had to attend a great number of people, and she said the nurses were never allowed to work on anything and take any responsible position, but that they had to be managed. And they had brought in the lay element. With all due respect to the Hon. Arthur Stanley and his interest in nurses, she said they had to call in lay members for management on the Council. They had tried to make a profession and they could not manage it—(laughter)—so they had brought in the laymen, and she doubted whether they would do any better under lay management than they had done under the General Medical Council. It was only when the nurses took upon themselves to manage their own Association and to work in their own interest and manage their own affairs that any Association, either the Royal College of Nursing or the Royal British Nurses' Association, could grow to any degree at all. (Hisses and slight applause.)

### The Value of the Word "College."

Dr. Stark Currie, who expressed the very deepest regret that there should be any dissentient voice there that day, said from his own experience, as a member of this Council for a considerable time, he could assure the lady who had just spoken that she was entirely mistaken and grievously mistaken in regard to some of her statements. He had never yet at any meetings of the Council which he had attended failed to see nurses, and representative nurses, and they had been allowed every freedom of speech. He hoped that there would be a very decided vote that day—an overwhelmingly decided vote—in favour of the scheme of amalgamation and co-operation. At first he was rather doubtful that it was a case of the lamb lying down with the lion, but of the lamb being inside the lion. He was convinced now that that was a mistaken view. The Royal British Association of Nurses would not lose its identity, much less lose its influence. It would carry its glory and honour into the new Association. The word "College" of itself carried great weight and influence. It would carry even more weight than the honoured title of the Royal British Nurses' Association, and he ventured to say even, with the utmost respect to his colleague Dr. Bedford Fenwick and Mrs. Bedford Fenwick, that it would carry more weight even than the Bedford Fenwick Association of Nurses. He saw a time coming when the noble profession of nursing would receive the status to which it had been too long entitled, and which it had not yet achieved, when the profession of surgeons, the profession of physicians, the profession of obstetricians, and the profession of nurses would stand on one common level, moving to one common goal—the good of humanity, the alleviation of suffering, and the welfare of mankind at large.

### The Oldest Living Nightingale Nurse.

Mrs. Dacre Craven, as the oldest living Nightingale nurse, said: If Miss Nightingale had lived to see that day she would have felt that the work she began had attained its highest fruition. The speaker hoped every person present would vote for the union of the Royal British Nurses' Association and the College of Nursing.

Miss Melrose said she had been a member of the Royal British Nurses' Association for a great number of years; she had been on the Executive for the past nine or ten years, and she hoped those present would vote solidly for the resolution.

Miss Little said when she came up from the North to the meeting she came with a certain amount of misgiving which Mr. Paterson had put quite to rest. The members of the Royal British Nurses' Association had always felt that the nurses' interests were safe in the hands of their Council, but there was a certain amount of jealousy and misgiving when she first heard of the proposed amalgamation with the College of Nursing. She would go back to the North with a good message for the nurses in her district, and they would feel from that time forth that that perhaps was the greatest day of all the days in the life of the Royal British Nurses' Association.

Miss Wortabet said about fifteen years ago she was in Paris, and saw the College there when it was started. She never saw anything more perfectly organised, or more perfectly under discipline, than she saw at that College. She thought it much better to have one big thing, one big organisation under one head. She thought a College would be a most splendid thing, and would respond to all their ideas and fulfil all their requirements.

### Did Dr. Griffith's Memory Fail Him?

Dr. Walter Griffith, as an old member of the Council and a life member of the Association, said he was very glad to hear of the proposed amalgamation. The original scheme was one which did not appeal to him, but the proposed amalgamation seemed likely to lead eventually to a successful result. He was not sure that he was in order in discussing the principles of the Charter—whether by agreeing to amalgamation they would also be considered to be agreeing to the Charter. He would also like to know whether the Charter, as maintained in the Journal, was in any sense approved by the meeting agreeing to the amalgamation.

The Chairman, in reply, said it would be open to anyone who wished to do so to make representations with regard to any alterations by sending them in either to the Executive Committee of the Royal British Nurses' Association, or, what was perhaps more direct, presenting them to the Privy Council when the Charter and by-laws came under consideration.

Dr. Griffith: The question was of principle, not of detail. It was what the relationship of that body, if constituted, would be to other bodies such as the Midwives Board, which was, of course, appointed by the State, and what relation the Council, which proposed to take the power to register all kinds of nurses, would have to bodies such as the Midwives Board. Further, was he correct in supposing that it was proposed that the new body should have the education of nurses in their hands—that they should decide on the education—that they should not only keep the Register but act as the Registrars, and, therefore, have powers comparable to those of the General Medical Council and of the Midwives Board?

The Chairman: I apprehend that the functions of the new body will never aspire in any way whatever to interfere with those of the Central Midwives Board. Speaking for myself—I am open to correction—the College of Nursing could not in any way enforce any system or standard of education until it had been recognised by Act of Parliament.

Dr. Griffith said he quite understood that, but did it not propose to attain those powers?

The Chairman did not think the College of Nursing would ever attempt to interfere. It would not interfere with other bodies in the slightest degree. In other respects he supposed they would take as much power as they could get.

Dr. Griffith: That is, of course, an answer, but not quite as clear an answer as I should like to have had. What I feel is this—that it is proposed and definitely said that the election of the governing body, that is the Council, shall be by the Corporation. That is the whole object. Is it likely or possible, if you want to obtain such powers, that you should put into the hands of us as we sit here the election of a Council with such enormous responsibilities and powers? Would they not be appointed in a

### THE ROYAL BRITISH NURSES' ASSOCIATION—(*continued*).

similar way to the Midwives Board and the General Medical Council?

Mrs. Bedford Fenwick: Hear, hear.

**NOTE.—Dr. Griffith and Mrs. Bedford Fenwick (?) would seem to have been unaware of the wording of the Nurses' Registration Bill 1916, which covers Dr. Griffith's point and provides for it. Clause 4 provides that the Council shall consist of representatives**

**(a) Of the Privy Council and any Government Department;**
**(b) of the Nurse-Training Schools;**
**(c) of the Medical Profession;**
**(d) of the Nurses on the Supplementary Registers; provided**
**(e) that not less than two-thirds of the Council shall be elected by the Nurses on the General Register under this Act.**

Dr. Griffith: It seems to me it is a matter for very serious consideration. I do not wish to say anything that would interfere with the proposed amalgamation. I am in favour of it, and I shall vote for it.

The Chairman: The College will not appoint the body; the body will be appointed by Act of Parliament. And I do not entertain any doubt personally, speaking for myself, that the Privy Council will retain for itself very considerable powers in relation to its functions.

### The Support of the Schools.

Mr. Comyns Berkeley then addressed the meeting in reference to the proposed amalgamation as a very old member of the Royal British Nurses' Association. He regarded the amalgamation as of vital importance to their interests. If it were carried through a large number of the most important matrons of England, Scotland, Wales, and, he hoped soon, Ireland would join their ranks, and with them would come the support of their hospitals and nursing schools. What had the members been doing all these years? They had been striving for three things—(1) for the State Registration of nurses; (2) apart from this, to improve the professional status of the trained nurse; and (3) for the welfare of the Association. They were among the first to agitate for the State Registration of trained nurses, and the Association had had several Bills before Parliament for that purpose. Since the foundation of the Royal British Nurses' Association the professional status of the trained nurse had been appreciably raised. And, lastly, the reputation of the Royal British Nurses' Association never stood higher than it did to-day. Throughout the whole of the English-speaking race it was known as the most important Association of trained nurses in the United Kingdom. It was his profound conviction that such an amalgamation as this would help in an inestimable way these three objects. He hoped that each member would be able to boast in years to come that she formed one of a large number of nurses who came from every part of England, Scotland, Ireland, and Wales, many at much pecuniary sacrifice, and most at great personal inconvenience, and passed a resolution which marked one of the greatest points in the history of nursing, to which trained nurses would be indebted for evermore.

The Chairman then put the resolution to the meeting. It was carried *nemine contradicente* amidst loud and prolonged applause.

### Votes of Thanks.

Mr. Comyns Berkeley then proposed a vote of thanks to the Chairman, Dr. Bezly Thorne, who, he said, was one of those who helped to found the Association, and from the first day till now had done every single thing he could to advance its interests. The vote was carried with loud applause.

In thanking the members, the Chairman said he congratulated them on the result of the meeting. He had the honour also to express to them the thanks of Her Royal Highness the President for the trouble they had taken in coming there that day. He did not suppose anyone had attended without some sacrifice of personal convenience, but when it was borne in mind that many present had come from the extreme parts of the kingdom, both North and South, that some had come from Scotland and some from Ireland in order to record their votes, they would understand that Her Royal Highness's thanks were not conventional or merely formal, but were very hearty indeed. Her Royal Highness also very specially appreciated the presence that afternoon of representatives of their great, prosperous, and ever-growing Australian branch. As to the part which Mr. Comyns Berkeley had taken from the very beginning in bringing about the fusion of these two bodies, he would like to say he had formed a link between them of the greatest possible value, and, but for him, the probability was that that meeting would never have taken place. Dr. Bezly Thorne also bore testimony to the unfailing courtesy, the consideration, the equity of mind, which had characterised everything that the Hon. Arthur Stanley had done and said in connection with the negotiations. The members might look upon the negotiations in the past as a courtship, upon the inestimable services of their legal adviser, Mr. Pitt, as being in the nature of preparing the marriage settlements, and the meeting as signalising a definite and formal betrothal. When the Privy Council had given its sanction to such changes as might seem desirable, they might look upon the nuptial knot as having been tied, and settle down together in happy domesticity to make themselves as useful as possible to the nursing profession and to the public at large.

A vote of thanks to Miss Macdonald, the Secretary, was then proposed by Mrs. Latter. Miss Macdonald, in reply, said she wished Mrs. Latter had told her she was going to do that very nice thing, because then she would have thought of something very nice to say to them. She would always remember how very kind the members had been to her as a Society. There were some people there that day whose friendship she very particularly appreciated, and those were the old nurses. She never knew people who liked so much to be called old. At first, when she came to the Association, she wondered why, but she soon found out that the reason was that they were the earliest members of the Royal British Nurses' Association.

After a vote of thanks to Mr. Paterson by Professor Glaister, the meeting, which was an enthusiastic one throughout, terminated.

